# Mitigation of biases in estimating hazard ratios under non-sensitive and non-specific observation of outcomes–applications to influenza vaccine effectiveness

**DOI:** 10.1186/s12982-020-00091-z

**Published:** 2021-01-14

**Authors:** Ulrike Baum, Sangita Kulathinal, Kari Auranen

**Affiliations:** 1 Department of Public Health Solutions, Finnish Institute for Health and Welfare, Mannerheimintie 166, 00300 Helsinki, Finland; 2 Department of Mathematics and Statistics, University of Helsinki, Helsinki, Finland; 3 Department of Mathematics and Statistics, University of Turku, Turku, Finland; 4 Department of Clinical Medicine, University of Turku, Turku, Finland

**Keywords:** Influenza, Outcome measurement error, Proportional hazards model, Survival analysis, Vaccine effectiveness

## Abstract

**Background:**

Non-sensitive and non-specific observation of outcomes in time-to-event data affects event counts as well as the risk sets, thus, biasing the estimation of hazard ratios. We investigate how imperfect observation of incident events affects the estimation of vaccine effectiveness based on hazard ratios.

**Methods:**

Imperfect time-to-event data contain two classes of events: a portion of the true events of interest; and false-positive events mistakenly recorded as events of interest. We develop an estimation method utilising a weighted partial likelihood and probabilistic deletion of false-positive events and assuming the sensitivity and the false-positive rate are known. The performance of the method is evaluated using simulated and Finnish register data.

**Results:**

The novel method enables unbiased semiparametric estimation of hazard ratios from imperfect time-to-event data. False-positive rates that are small can be approximated to be zero without inducing bias. The method is robust to misspecification of the sensitivity as long as the ratio of the sensitivity in the vaccinated and the unvaccinated is specified correctly and the cumulative risk of the true event is small.

**Conclusions:**

The weighted partial likelihood can be used to adjust for outcome measurement errors in the estimation of hazard ratios and effectiveness but requires specifying the sensitivity and the false-positive rate. In absence of exact information about these parameters, the method works as a tool for assessing the potential magnitude of bias given a range of likely parameter values.

## Introduction

Outcome measurement errors are common in epidemiological studies and may bias the estimated effects of exposures or interventions on health outcomes. When a binary outcome such as presence/absence of infection is measured with error, the problem is called outcome misclassification [1]. The impact of outcome misclassification on estimation of risk ratios has been studied thoroughly [2–4]. Nevertheless, the same lessons cannot be readily adopted when estimating hazard ratios from time-to-event data because imperfectly observed event times do not only affect event counts but may also bias the at-risk times and thus the risk set sizes.

A particular problem arises when estimating vaccine effectiveness as the relative reduction in the infection hazard. If infection-induced immunity reduces or removes the risk of subsequent infection with the same pathogen, non-sensitive measurement of infection inflates the risk set. For example, influenza is likely to immunise the human host at least temporarily and all infections in a large population are never recorded in practice. Moreover, false-positive records may occur due to imperfect specificity of diagnostic procedures.

Yang et al. [5] addressed estimation of vaccine effectiveness under non-specific observation of influenza infection using a subset of acute respiratory infections as a validation set on disease aetiology. An expectation–maximisation algorithm was developed to account for the uncertainty in the aetiology of infections outside the validation set [5]. Although the validation data carried information on the specificity, perfect sensitivity was assumed.

Meier et al. [6] focused on detection of chronic outcomes such as human immunodeficiency virus infection, which if initially missed can still be detected by later testing. A full-likelihood approach was developed to estimate the hazard ratio under repeated usage of an imperfect laboratory test, based on a proportional hazards (PH) model in discrete time and assuming the test sensitivity and specificity are known [6]. However, this method cannot be applied under imperfect observation of incident events, such as influenza infection, which by standard laboratory tests can only be detected up to one week after symptom onset [7].

The role of non-sensitive and non-specific observation of incident infection outcomes on the estimation of hazard ratios has thus not been fully covered in previous literature. We here study how outcome measurement errors affect the estimation of vaccine effectiveness based on hazard ratios. We modify the standard partial likelihood under the PH model [8] to adjust for outcome measurement errors in time-to-event data, assuming the sensitivity and the false-positive rate are known. We explore the magnitude of bias when the measurement errors are not corrected for and evaluate the robustness of effectiveness estimates to misspecification of the sensitivity and the false-positive rate. We implement the new method in R [9] (see Additional file 1: R script) and use simulated and Finnish register data to show its performance. Our work is motivated by the Finnish policy of estimating influenza vaccine effectiveness each season from register data [10], which do not include influenza-negative test results and thus do not allow for a retrospective design such as the widely used test-negative design [11]. Therefore, we here focus exclusively on cohort studies.

## Methods

### True and false-positive events

We consider the sensitivity of outcome measurement as the conditional probability for the true event of interest being recorded in the data. When the sensitivity is less than 1, some true events may not be recorded. Additionally, other events may be mistakenly recorded as events of interest. Such false-positive records make outcome measurement non-specific. Here, the true event means influenza infection while false-positive events are any other (non-influenza) events incorrectly recorded as influenza.

**Fig. 1:**
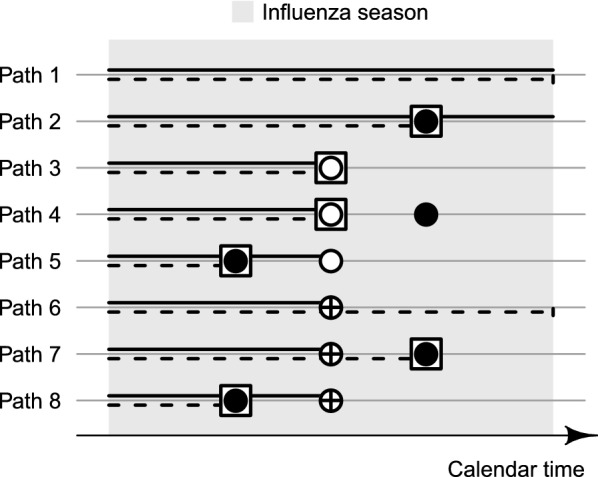
Occurrence of true and false-positive influenza events. The figure shows the eight possible paths of events for a study subject during the study period (influenza season). The true event is depicted either by a white circle if it was recorded or by a crossed circle if it was not recorded. False-positive events are depicted by black circles. Although false positives may occur repeatedly, the figure shows only the first of these if any. The subject’s true time at risk during the study period is marked by a solid line. In the study, the subject’s follow-up (dashed line) ends at the time of the first recorded event, which is highlighted by a square around the event-defining circle, or at the time of censoring (vertical bar). Although recorded, the true event is not part of the data under study if there is a preceding false-positive event. The true at-risk time is then underestimated (Paths 2, 5 and 8). By contrast, Paths 6 and 7 show scenarios in which the true at-risk time is overestimated

For any one subject, let the hazard of the true event at time $t$ be $\lambda (t)$. We assume that the true event occurs at most once during the study period, and if it occurs, is recorded with sensitivity $se$. False-positive events may occur repeatedly at rate $\kappa (t)$. Originally, the data may thus comprise more than one event per subject. In the study, however, based on the assumption that the true event is unique, each subject’s follow-up is set to end at his/her first recorded event or censoring, whichever occurs first. The data under study therefore comprise at most one recorded event per subject (Fig. 1). The event’s status as true or false positive is indistinguishable by observation.

If the true event occurs but is not recorded, the subject’s follow-up continues beyond the true event time, erroneously lengthening the at-risk time in the study. By contrast, should a false-positive event occur before the true event, the subject’s follow-up ends prematurely and the true event time is not part of the data under study (Fig. 1). This also shows that minor violations of the above assumption of uniqueness would not compromise the study.

### True and observed survival functions

The true survival function $S(t)$ is the probability to escape the true event beyond time $t$. The observed survival function $\tilde{S}(t)$ is the probability to avoid detection of the true event and the occurrence of any false-positive events beyond $t$. Assuming constant sensitivity ($se$) and false-positive rate ($\kappa$), the relationship between the survival functions is (cf. Additional file 2: Web Appendix)

1 $$\tilde{S}(t;se,\;\kappa ) = [1 - se \cdot (1 - S(t))] \cdot e^{( - \kappa t)} .$$

The first right-hand-side term is the complement probability of the true event having occurred and been recorded by $t$. The second term is the probability of no false-positive events having occurred by $t$.

### True and observed hazards

Given expression (1), the relation between the hazard $\tilde{\lambda}(t)$ of the recorded event (observed hazard) and the hazard $\lambda (t)$ of the true event (true hazard) follows (cf. Additional file 2: Web Appendix):

2 $$\tilde{\lambda}\left(t;se,\kappa \right)=se\cdot \frac{S\left(t\right)}{\tilde{S}\left(t; se,\kappa =0\right)}\cdot \lambda \left(t\right)+\kappa =se \cdot w(t)\cdot \lambda \left(t\right)+\kappa,$$

where the weight $w(t)$ is defined as

$$w\left( t \right) = \frac{S\left( t \right)}{{\tilde{S}\left( {t; se,\kappa = 0} \right)}}.$$

The observed hazard is thus the sum of the hazard of recording the true event and the false-positive rate. The sensitivity $se$ accounts for the possibility of not recording the true event. The weight $w(t)$ equals the ratio of the true survival probability and the survival probability observed in absence of false positives and adjusts for the fact that a true but unrecorded event may have already removed the subject from the study’s risk set before time $t$. It holds that $w(t)\le 1$.

### Vaccine effectiveness

We compare the true hazards between two groups defined by vaccination as binary exposure. Specifically, ${\lambda }_{0}(t)$ and ${\lambda }_{1}(t)$ denote the true hazards for unvaccinated and vaccinated subjects, respectively, as functions of time since season onset. The estimand of interest is vaccine effectiveness ($VE$) defined as the relative reduction in the infection hazard [12]:

$$VE\left( t \right) = 1 - \frac{{\lambda_{1} \left( t \right)}}{{\lambda_{0} \left( t \right)}}.$$

In this paper, the true hazards in unvaccinated and vaccinated subjects are assumed to be proportional over time so that the $VE$ estimand is constant. If $\kappa =0$, it follows from (2) that

3 $${\tilde{\lambda }}_{v}\left(t;se,\kappa =0\right)={(1-VE)}^{v}\cdot {se}_{v}\cdot {w}_{v}(t)\cdot {\lambda }_{0}\left(t\right),$$

where

$$w_{v} \left( t \right) = \frac{{S_{v} \left( t \right)}}{{\tilde{S}_{v} \left( {t; se_{v} ,\kappa = 0} \right)}}$$

and $v=0$ (unvaccinated) or $1$ (vaccinated).

### Weighted partial likelihood under imperfect sensitivity in absence of false positives

The data under study comprise $n$ recorded events in a cohort of unvaccinated and vaccinated subjects. The event times of the $n$ cases are ${{t}_{1}<t}_{2}<\dots <{t}_{n}$. Let ${\tilde{N}}_{0}({t}_{i})$ and ${\tilde{N}}_{1}({t}_{i})$ denote the numbers of unvaccinated and vaccinated subjects in the risk set at ${t}_{i}$. Of note, a subject’s vaccination status may change over time [10].

Under the PH assumption, the hazard ratio and thus $VE$ can be estimated from complete and perfectly measured time-to-event data by maximising the standard partial likelihood [8]. Here, we adjust the partial likelihood to allow estimation of $VE$ under imperfect sensitivity. When ${se}_{0}$ and ${se}_{1}$ are known, the partial likelihood of $VE$ is.

4 $$L\left(VE\right)=\prod_{i=1}^{n}{L}_{i}\left(VE\right) =\prod_{i=1}^{n}\left[{\tilde{\lambda }}_{{v}_{i}}({t}_{i})/\sum_{j=0}^{1}\left({\tilde{\lambda }}_{j}\left({t}_{i}\right){\cdot \tilde{N}}_{j}\left({t}_{i}\right)\right)\right],$$

where ${L}_{i}(VE)$ is the conditional probability for the event occurring to case $i$ given the risk set at ${t}_{i}$, and ${v}_{i}$ is 0 if case $i$ is unvaccinated and 1 otherwise. Using (3), ${L}_{i}(VE)$ is obtained as

5 $${L}_{i}\left(VE\right)=\frac{{\left(1-VE\right)}^{{v}_{i}}\cdot {se}_{{v}_{i}}\cdot {w}_{{v}_{i}}\left({t}_{i}\right)}{{se}_{0}\cdot {w}_{0}\left({t}_{i}\right)\cdot {\tilde{N}}_{0}\left({t}_{i}\right)+{\left(1-VE\right)\cdot se}_{1}\cdot {w}_{1}\left({t}_{i}\right)\cdot {\tilde{N}}_{1}\left({t}_{i}\right)}, i=1,\dots ,n.$$

Unlike the standard partial likelihood, (5) depends on weights ${w}_{0}(t)$ and ${w}_{1}(t)$, which correct for the too large risk set following from imperfect sensitivity. Using the Kaplan–Meier estimate $\widehat{\tilde{S}}(t)$ for $\tilde{S}(t;se,\kappa =0)$ leads to plug-in weights ${\widehat{w}}_{0}(t)$ and ${\widehat{w}}_{1}(t)$ (cf. Additional file 2: Web Appendix):

6 $${\widehat{w}}_{v}\left(t\right)=\frac{1-(1-{\widehat{\tilde{S}}}_{v}(t))/{se}_{v}}{{\widehat{\tilde{S}}}_{v}\left(t\right)}, v\in \{\mathrm{0,1}\}.$$

$VE$ is estimated by maximising (4) and its standard error ($SE$) can be obtained using the Fisher information. If ${se}_{0}={se}_{1}=1$, (4) simplifies to the standard partial likelihood.

### Probabilistic deletion of false-positive events

If false-positive events occur, i.e. if $\kappa >0$, semiparametric estimation using the weighted partial likelihood is not directly applicable as the true hazard ${\lambda }_{0}(t)$ does not cancel out affecting expression (5). We propose an approach that retains only a portion of the $n$ recorded events by approximating the time-varying probability of the recorded event being a true event.

The probability that an event observed at time ${t}_{i}$ is a true event is given by the ratio of the hazard of recording the true event to the observed hazard. This probability is (cf. Additional file 2: Web Appendix)

7 $${p}_{v}\left({t}_{i}\right)=\frac{{\tilde{\lambda }}_{v}\left({t}_{i}\right)-\kappa }{{\tilde{\lambda }}_{v}\left({t}_{i}\right)}, v\in \{\mathrm{0,1}\}.$$

We suggest approximating ${\tilde{\lambda }}_{v}\left({t}_{i}\right)$ over a short time window ($\Delta {t}_{i}$) centred around ${t}_{i}$ as the number of events observed (${\tilde{D}}_{v,i}$) per person-time ($\tilde N_v(t_i)\cdot {\Delta t}_{i}$) and, hence, an approximation to ${p}_{v}({t}_{i})$ is given by

8 $${p}_{v}\left({t}_{i}\right)\approx \frac{{\tilde{D}}_{v,i}/({\tilde{N}}_{v}({t}_{i})\cdot \Delta {t}_{i})-\kappa }{{\tilde{D}}_{v,i}/({\tilde{N}}_{v}({t}_{i})\cdot \Delta {t}_{i})}.$$

Subsequently, any event observed at ${t}_{i}$ is retained in the data with probability ${p}_{v}({t}_{i})$, corresponding to censoring events at each ${t}_{i}$ with probability $1-{p}_{v}({t}_{i})$. In analogy to multiple imputation, the above procedure is repeated a number of times to produce replicate data sets. Each resulting set of time-to-event data is analysed as in absence of false positives using the weighted partial likelihood. At the end, the $VE$ estimates are pooled taking into account the within- and the between-imputation variability [13].

## Simulation study

### Set-up

We conducted a simulation study to assess the performance of the proposed methods. Briefly, true event times were simulated according to hazards ${\lambda }_{0}\left(t\right)$ (unvaccinated) and ${\left(1-VE\right)\cdot \lambda }_{0}(t)$ (vaccinated), where ${\lambda }_{0}\left(t\right)$ mimicked the force of infection in a Susceptible-Infected-Removed epidemic [14] with cumulative risk of 0.25 (alternatively 0.81) over a 196-day influenza season (cf. Additional file 2: Web Appendix). Two separate cohorts were considered, comprising 50,000 (30% vaccinated at season onset) and 1,000,000 (50% vaccinated) individuals, corresponding to the cohort sizes of Finnish children and elderly, respectively [10, 15, 16]. $VE$ was 10%, 30%, 50%, 70% or 90%.

For each individual, observed true events were realised by retaining simulated true events with sensitivities ${se}_{0}$ (unvaccinated) and ${se}_{1}$ (vaccinated). Values ${se}_{0}={se}_{1}=0.04$ were based on a Finnish study of the 2009/10 influenza season [17]. Alternatively, values ${se}_{0}=0.05$ and ${se}_{1}=0.03$ were employed to investigate differential sensitivity. A false-positive event time was sampled from the exponential distribution with rates corresponding to 2% or 16% of all recorded events in the unvaccinated being false-positive. The smaller of the observed true and false-positive event times was used as the recorded event time for the individual.

For each setting (cohort size, $VE$, ${se}_{0}$, ${se}_{1}$ and $\kappa$), 10^4^ repeated datasets were simulated. For each dataset, ten random subsets were created by retaining events with probability $p(t)$ as in (8). Adjusted $VE$ estimates were computed with the same values of ${se}_{0}$, ${se}_{1}$ and $\kappa$ as used in simulation. In addition, naïve $VE$ estimates were obtained by incorrectly assuming perfect sensitivity (${se}_{0}={se}_{1}=1$) and/or absence of false positives ($\kappa =0$). Finally, the ten dataset-specific estimates were pooled resulting in 10^4^ estimates of $VE$ and $SE$ per setting.

We report the bias as the difference between the mean of $VE$ estimates ($\widehat{VE}$) and the true $VE$. We compare the mean of the $SE$ estimates ($\widehat{SE}$) with the empirical standard error of the $VE$ estimates (${SE}_{\widehat{VE}}$). The estimation error (${\sqrt{MSE}}_{\widehat{VE}}$) is assessed as the root-mean-squared error between the $VE$ estimates and the true $VE$. The empirical coverage probability of the 95% confidence interval (CI) was estimated as the percentage of 10^4^ CIs that included the true $VE$.

### Estimation of vaccine effectiveness under imperfect sensitivity in absence of false-positive events

Tables 1 and 2 show the adjusted and naïve $VE$ estimates under non-differential sensitivity (${se}_{0}={se}_{1}=0.04$) and differential sensitivity (${se}_{0}=0.05$, ${se}_{1}=0.03$), respectively, with $\kappa =0$ and cumulative risk of 0.25 in the unvaccinated. Table 3 and Additional file 2: Table S1 (see Additional file 2: Web Appendix) show the corresponding estimates under cumulative risk of 0.81.

**Table 1:** Estimates of vaccine effectiveness (${\varvec{V}}{\varvec{E}}$) under non-differentially imperfect sensitivity and small cumulative risk of infection in absence of false-positive events

True	Estimation adjusted for ${se}_{0}={se}_{1}=0.04$						Naïve estimation					
$VE$	$\widehat{VE}$	$\widehat{SE}$	${SE}_{\widehat{VE}}$	${\sqrt{MSE}}_{\widehat{VE}}$	Bias	Cov	$\widehat{VE}$	$\widehat{SE}$	${SE}_{\widehat{VE}}$	${\sqrt{MSE}}_{\widehat{VE}}$	Bias	Cov
Cohort of 50,000 individuals (30% vaccinated at season onset)												
10%	10%	0.09	0.10	0.10	± 0	92%	9%	0.09	0.09	0.09	− 1	95%
30%	30%	0.08	0.09	0.09	± 0	92%	27%	0.08	0.08	0.09	− 3	95%
50%	50%	0.06	0.07	0.07	± 0	93%	46%	0.07	0.07	0.08	− 4	94%
70%	70%	0.05	0.05	0.05	± 0	93%	67%	0.05	0.05	0.06	− 3	93%
90%	90%	0.02	0.03	0.03	± 0	94%	89%	0.03	0.03	0.03	− 1	95%
Cohort of 1,000,000 individuals (50% vaccinated at season onset)												
10%	10%	0.02	0.02	0.02	± 0	92%	9%	0.02	0.02	0.02	− 1	91%
30%	30%	0.02	0.02	0.02	± 0	91%	27%	0.02	0.02	0.03	− 3	57%
50%	50%	0.01	0.01	0.01	± 0	93%	47%	0.01	0.01	0.04	− 3	23%
70%	70%	0.01	0.01	0.01	± 0	94%	67%	0.01	0.01	0.03	− 3	10%
90%	90%	0.00	0.00	0.00	± 0	94%	89%	0.01	0.01	0.01	− 1	26%

Mean of the vaccine effectiveness estimates ($\widehat{VE}$), mean of the standard error estimates ($\widehat{SE}$), standard error of the vaccine effectiveness estimates (${SE}_{\widehat{VE}}$), root-mean-squared error of the vaccine effectiveness estimates (${\sqrt{MSE}}_{\widehat{VE}}$), bias in percentage points, and empirical coverage probability (Cov) of the 95% confidence intervals when estimating vaccine effectiveness from 10^4^ repeated data sets under non-differential sensitivity (${se}_{0}={se}_{1}$) of 0.04 and a cumulative risk of 0.25 in the unvaccinated in absence of false-positive events. Naïve estimation was conducted under the incorrect assumption of perfect sensitivity (${se}_{0}={se}_{1}=1$)

**Table 2:** Estimates of vaccine effectiveness (${\varvec{V}}{\varvec{E}}$) under differential sensitivity and small cumulative risk of infection in absence of false-positive events

True	Estimation adjusted for ${se}_{0}=0.05$, ${se}_{1}=0.03$						Naïve estimation					
$VE$	$\widehat{VE}$	$\widehat{SE}$	${SE}_{\widehat{VE}}$	${\sqrt{MSE}}_{\widehat{VE}}$	Bias	Cov	$\widehat{VE}$	$\widehat{SE}$	${SE}_{\widehat{VE}}$	${\sqrt{MSE}}_{\widehat{VE}}$	Bias	Cov
Cohort of 50,000 individuals (30% vaccinated at season onset)												
10%	10%	0.10	0.11	0.11	± 0	91%	45%	0.06	0.06	0.36	+ 35	0%
30%	30%	0.08	0.09	0.09	± 0	92%	56%	0.05	0.05	0.27	+ 26	1%
50%	50%	0.07	0.08	0.08	± 0	92%	68%	0.04	0.04	0.19	+ 18	4%
70%	70%	0.05	0.05	0.05	± 0	93%	80%	0.03	0.03	0.11	+ 10	19%
90%	90%	0.03	0.03	0.03	± 0	93%	93%	0.02	0.02	0.04	+ 3	57%
Cohort of 1,000,000 individuals (50% vaccinated at season onset)												
10%	10%	0.02	0.02	0.02	± 0	92%	45%	0.01	0.01	0.35	+ 35	0%
30%	30%	0.02	0.02	0.02	± 0	91%	56%	0.01	0.01	0.26	+ 26	0%
50%	50%	0.01	0.01	0.01	± 0	93%	68%	0.01	0.01	0.18	+ 18	0%
70%	70%	0.01	0.01	0.01	± 0	94%	80%	0.01	0.01	0.10	+ 10	0%
90%	90%	0.01	0.01	0.01	± 0	94%	93%	0.00	0.00	0.03	+ 3	0%

Mean of the vaccine effectiveness estimates ($\widehat{VE}$), mean of the standard error estimates ($\widehat{SE}$), standard error of the vaccine effectiveness estimates (${SE}_{\widehat{VE}}$), root-mean-squared error of the vaccine effectiveness estimates (${\sqrt{MSE}}_{\widehat{VE}}$), bias in percentage points, and empirical coverage probability (Cov) of the 95% confidence intervals when estimating vaccine effectiveness from 10^4^ repeated data sets under differential sensitivity of 0.05 (${se}_{0}$) and 0.03 (${se}_{1}$) and a cumulative risk of 0.25 in the unvaccinated in absence of false-positive events. Naïve estimation was conducted under the incorrect assumption of perfect sensitivity (${se}_{0}={se}_{1}=1$)

**Table 3:** Estimates of vaccine effectiveness (${\varvec{V}}{\varvec{E}}$) under non-differentially imperfect sensitivity and high cumulative risk of infection in absence of false-positive events

True	Estimation adjusted for ${se}_{0}={se}_{1}=0.04$						Naïve estimation					
$VE$	$\widehat{VE}$	$\widehat{SE}$	${SE}_{\widehat{VE}}$	${\sqrt{MSE}}_{\widehat{VE}}$	Bias	Cov	$\widehat{VE}$	$\widehat{SE}$	${SE}_{\widehat{VE}}$	${\sqrt{MSE}}_{\widehat{VE}}$	Bias	Cov
Cohort of 50,000 individuals (30% vaccinated at season onset)												
10%	10%	0.05	0.10	0.10	± 0	67%	4%	0.05	0.05	0.08	− 6	82%
30%	30%	0.04	0.08	0.08	± 0	70%	15%	0.05	0.05	0.16	− 15	11%
50%	50%	0.03	0.05	0.05	± 0	75%	30%	0.04	0.04	0.20	− 20	0%
70%	70%	0.02	0.03	0.03	± 0	80%	51%	0.03	0.03	0.19	− 19	0%
90%	90%	0.01	0.01	0.01	± 0	88%	81%	0.02	0.02	0.09	− 9	0%
Cohort of 1,000,000 individuals (50% vaccinated at season onset)												
10%	10%	0.01	0.02	0.02	± 0	66%	4%	0.01	0.01	0.06	− 6	0%
30%	30%	0.01	0.02	0.02	± 0	69%	15%	0.01	0.01	0.15	− 15	0%
50%	50%	0.01	0.01	0.01	± 0	71%	30%	0.01	0.01	0.20	− 20	0%
70%	70%	0.00	0.01	0.01	± 0	75%	52%	0.01	0.01	0.18	− 18	0%
90%	90%	0.00	0.00	0.00	± 0	83%	81%	0.00	0.00	0.09	− 9	0%

Mean of the vaccine effectiveness estimates ($\widehat{VE}$), mean of the standard error estimates ($\widehat{SE}$), standard error of the vaccine effectiveness estimates (${SE}_{\widehat{VE}}$), root-mean-squared error of the vaccine effectiveness estimates (${\sqrt{MSE}}_{\widehat{VE}}$), bias in percentage points, and empirical coverage probability (Cov) of the 95% confidence intervals when estimating vaccine effectiveness from 10^4^ repeated data sets under non-differential sensitivity (${se}_{0}={se}_{1}$) of 0.04 and a cumulative risk of 0.81 in the unvaccinated in absence of false-positive events. Naïve estimation was conducted under the incorrect assumption of perfect sensitivity (${se}_{0}={se}_{1}=1$)

The adjusted $\widehat{VE}$’s are unbiased. Therefore, the estimation error is equal to the standard error (${\sqrt{MSE}}_{\widehat{VE}}={SE}_{\widehat{VE}}$). Because the uncertainty in the plug-in weights is not taken into account, the standard errors are slightly underestimated ($\widehat{SE}<{SE}_{\widehat{VE}}$), leading to smaller than nominal CI coverage probabilities.

Under non-differential sensitivity, the naïve $\widehat{VE}$’s underestimate the true $VE$. The bias is stronger when the cumulative risk is high (0.81). When the cumulative risk is small (0.25), the estimation errors in the naïve and adjusted estimates are similar. However, as standard errors may be small, even slight biases can lead to poor CI coverage. Under differential sensitivity (${se}_{0}>{se}_{1}$) and small cumulative risk, the naïve $\widehat{VE}$’s overestimate the true $VE$. The estimation error in the naïve estimates exceeds the one in the adjusted estimates indicating that the estimation is not robust to gross misspecification of ${se}_{0}$ and ${se}_{1}$. The error attenuates when the cumulative risk is high.

### Estimation of vaccine effectiveness under imperfect sensitivity and false-positive events

Table 4 and Additional file 2: Table S2 (see Additional file 2: Web Appendix) show the adjusted and naïve $VE$ estimates under non-differential sensitivity (${se}_{0}={se}_{1}=0.04$) and differential sensitivity (${se}_{0}=0.05$, ${se}_{1}=0.03$), respectively, with cumulative risk of 0.25 and false-positive proportion of 2% among unvaccinated. In general, the results correspond to the situation without false positives. The adjusted $\widehat{VE}$’s are essentially unbiased. As the naïve $\widehat{VE}$’s do not differ much between settings with and without false positives, a false-positive rate corresponding to a false-positive proportion of 2% among unvaccinated does not essentially affect the estimation. However, under the higher false-positive proportion (16%) naïve estimates are biased but adjusted estimation performs well (Table 5).

**Table 4:** Estimates of vaccine effectiveness (${\varvec{V}}{\varvec{E}}$) under non-differentially imperfect sensitivity, small cumulative risk of infection and low rate of false-positive events

True	Estimation adjusted for ${se}_{0}={se}_{1}=0.04$, $\kappa ={10}^{-6}$						Naïve estimation					
$VE$	$\widehat{VE}$	$\widehat{SE}$	${SE}_{\widehat{VE}}$	${\sqrt{MSE}}_{\widehat{VE}}$	Bias	Cov	$\widehat{VE}$	$\widehat{SE}$	${SE}_{\widehat{VE}}$	${\sqrt{MSE}}_{\widehat{VE}}$	Bias	Cov
Cohort of 50,000 individuals (30% vaccinated at season onset)												
10%	10%	0.09	0.11	0.11	± 0	92%	8%	0.09	0.09	0.09	− 2	95%
30%	30%	0.08	0.09	0.09	± 0	92%	26%	0.08	0.08	0.09	− 4	95%
50%	50%	0.06	0.07	0.07	± 0	93%	46%	0.07	0.07	0.08	− 4	93%
70%	69%	0.05	0.05	0.05	− 1	94%	66%	0.05	0.05	0.07	− 4	90%
90%	89%	0.03	0.03	0.03	− 1	96%	87%	0.03	0.03	0.04	− 3	88%
Cohort of 1,000,000 individuals (50% vaccinated at season onset)												
10%	10%	0.02	0.02	0.02	± 0	92%	9%	0.02	0.02	0.02	− 1	89%
30%	30%	0.02	0.02	0.02	± 0	91%	27%	0.02	0.02	0.04	− 3	44%
50%	50%	0.01	0.01	0.01	± 0	93%	46%	0.01	0.01	0.05	− 4	7%
70%	70%	0.01	0.01	0.01	± 0	94%	66%	0.01	0.01	0.04	− 4	0%
90%	90%	0.00	0.00	0.00	± 0	94%	87%	0.01	0.01	0.03	− 3	0%

Mean of the vaccine effectiveness estimates ($\widehat{VE}$), mean of the standard error estimates ($\widehat{SE}$), standard error of the vaccine effectiveness estimates (${SE}_{\widehat{VE}}$), root-mean-squared error of the vaccine effectiveness estimates (${\sqrt{MSE}}_{\widehat{VE}}$), bias in percentage points, and empirical coverage probability (Cov) of the 95% confidence intervals when estimating vaccine effectiveness from 10^4^ repeated data sets given non-differential sensitivity (${se}_{0}={se}_{1}$) of 0.04 and a cumulative risk of 0.25 in the unvaccinated. The false-positive events occurred at rate $\kappa ={10}^{-6}$ (per person-day) corresponding to a false-positive proportion of 2% among the unvaccinated. Naïve estimation was conducted under the incorrect assumptions of perfect sensitivity (${se}_{0}={se}_{1}=1$) and absence of false positives ($\kappa =0$)

**Table 5:** Estimates of vaccine effectiveness (${\varvec{V}}{\varvec{E}}$) under non-differentially imperfect sensitivity, small cumulative risk of infection and high rate of false-positive events

True	Estimation adjusted for ${se}_{0}={se}_{1}=0.04$, $\kappa ={10}^{-5}$						Naïve estimation					
$VE$	$\widehat{VE}$	$\widehat{SE}$	${SE}_{\widehat{VE}}$	${\sqrt{MSE}}_{\widehat{VE}}$	Bias	Cov	$\widehat{VE}$	$\widehat{SE}$	${SE}_{\widehat{VE}}$	${\sqrt{MSE}}_{\widehat{VE}}$	Bias	Cov
Cohort of 50,000 individuals (30% vaccinated at season onset)												
10%	9%	0.10	0.11	0.12	− 1	92%	7%	0.09	0.09	0.09	− 3	95%
30%	29%	0.09	0.10	0.10	− 1	92%	23%	0.08	0.08	0.11	− 7	87%
50%	49%	0.07	0.08	0.08	− 1	93%	39%	0.07	0.07	0.13	− 11	63%
70%	68%	0.05	0.06	0.06	− 2	93%	56%	0.05	0.05	0.15	− 14	23%
90%	86%	0.04	0.04	0.05	− 4	85%	74%	0.04	0.04	0.17	− 16	0%
Cohort of 1,000,000 individuals (50% vaccinated at season onset)												
10%	10%	0.02	0.02	0.02	± 0	92%	7%	0.02	0.02	0.03	− 3	67%
30%	30%	0.02	0.02	0.02	± 0	91%	23%	0.02	0.02	0.07	− 7	0%
50%	50%	0.01	0.01	0.01	± 0	92%	39%	0.01	0.01	0.11	− 11	0%
70%	70%	0.01	0.01	0.01	± 0	92%	56%	0.01	0.01	0.14	− 14	0%
90%	90%	0.01	0.01	0.01	± 0	87%	74%	0.01	0.01	0.16	− 16	0%

Mean of the vaccine effectiveness estimates ($\widehat{VE}$), mean of the standard error estimates ($\widehat{SE}$), standard error of the vaccine effectiveness estimates (${SE}_{\widehat{VE}}$), root-mean-squared error of the vaccine effectiveness estimates (${\sqrt{MSE}}_{\widehat{VE}}$), bias in percentage points, and empirical coverage probability (Cov) of the 95% confidence intervals when estimating vaccine effectiveness from 10^4^ repeated data sets given non-differential sensitivity (${se}_{0}={se}_{1}$) of 0.04 and a cumulative risk of 0.25 in the unvaccinated. The false-positive events occurred at rate $\kappa ={10}^{-5}$ (per person-day) corresponding to a false-positive proportion of 16% among the unvaccinated. Naïve estimation was conducted under the incorrect assumptions of perfect sensitivity (${se}_{0}={se}_{1}=1$) and absence of false positives ($\kappa =0$)

### Influenza vaccine effectiveness in the Finnish elderly

This section presents estimates of influenza vaccine effectiveness in Finnish elderly in 2016/17, a season dominated by influenza subtype A/H3N2. Briefly, a nationwide cohort of individuals aged 65 years and above was monitored through the season (196 days), using data collected as part of healthcare routines. The outcome was laboratory-confirmed influenza, which is a non-sensitive measurement of influenza infection as not everyone seeks healthcare or is swabbed. The register-based cohort study design is described in detail elsewhere [10]. For simplicity, we here focus on outcome measurement errors assuming absence of other sources of bias such as exposure measurement errors or confounding.

The cohort totalled 1,160,986 individuals of which 47% were vaccinated during the season. There were 8389 recorded events of which 3346 occurred in vaccinated individuals. Unlike in the simulation study, $VE$, ${se}_{0}$, ${se}_{1}$ and $\kappa$ were unknown. The sensitivities (${se}_{0}$, ${se}_{1}$) were set at 0.04 (cf. Shubin et al. [17]). The false-positive rate ($\kappa$) was deemed very small and thus approximated as 0.

**Fig. 2:**
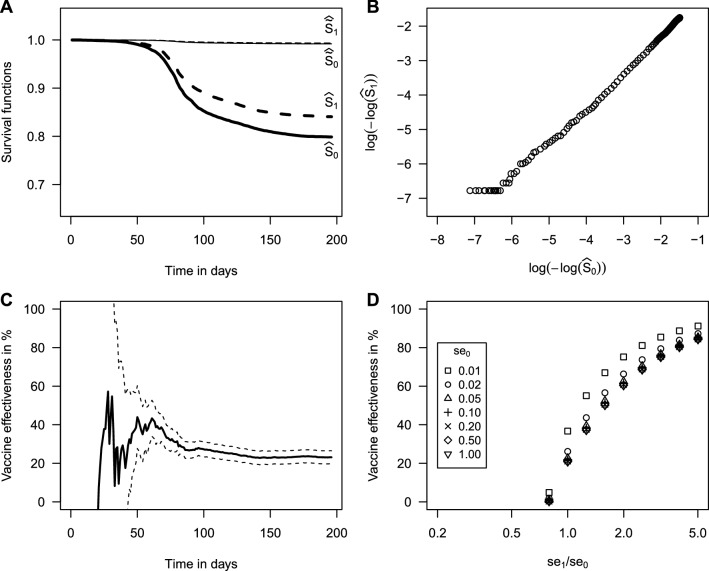
Estimates of influenza vaccine effectiveness in the Finnish elderly (N = 1,160,986) in 2016/17. A: Kaplan–Meier estimates of the observed survival functions in the unvaccinated (${\widehat{\tilde{S}}}_{0}(t)$) and vaccinated (${\widehat{\tilde{S}}}_{1}(t)$) and the corresponding estimates of the true survival functions (${\widehat{S}}_{0}(t)$, ${\widehat{S}}_{1}(t)$) based on (1) assuming non-differential sensitivity (${se}_{0}$, ${se}_{1}$) of 0.04 and absence of false-positive events. The estimated cumulative risks ($1-{\widehat{S}}_{0}(t)$, $1-{\widehat{S}}_{1}(t)$) at $t=196$ (days) were 0.20 and 0.16. B: The linear relation between the log–log transformed survival functions ${\widehat{S}}_{0}(t)$ and ${\widehat{S}}_{1}(t)$ supports the proportional hazards assumption (cf. Additional file 2: Web Appendix). C: Time evolution of vaccine effectiveness estimates (solid line) and pointwise 95% confidence intervals (dashed lines) based on (4). D: Dependence of vaccine effectiveness estimates at $t=196$ (days) on the assumed values of ${se}_{0}$ (symbols) and ratio ${se}_{1}/{se}_{0}$ (horizontal axis) based on (4). The plot area has been restricted showing only non-negative vaccine effectiveness estimates. For the full range see Additional file 2: Figure S1 (see Additional file 2: Web Appendix)

The estimated cumulative risks over the season were 0.20 (unvaccinated) and 0.16 (vaccinated; Fig. 2a). The linear relation between the log–log transformed survival functions supports the PH assumption (Fig. 2b). The adjusted $VE$ estimate at $t=196$ (days) was 23% (95% CI 20–26%; Fig. 2c) similar to the naïve (${se}_{0}={se}_{1}=1$) estimate 21% (95% CI 17–24%; Fig. 2d).

The estimates were mainly affected by the ratio ${se}_{1}/{se}_{0}$ unless ${se}_{0}$ was chosen very small (Fig. 2d). Assuming ${se}_{0}={se}_{1}=0.01$, for instance, implied rather unrealistic cumulative risks of 0.81 (unvaccinated) and 0.64 (vaccinated). The corresponding $VE$ estimate was 37% (95% CI 34%–39%). If vaccinated cases were assumed to be less likely detected (${se}_{1}/{se}_{0}<1$), the $VE$ estimates were smaller than 23%, and vice versa. For example, if ${se}_{1}=0.04$ and ${se}_{0}=0.05$, $VE$ was 2% with 95% CI from –3% to 6%, indicating that vaccination may not have been effective.

## Discussion

Motivated by the Finnish policy of evaluating influenza vaccine effectiveness from register data, we developed a weighted partial likelihood approach with probabilistic deletion of false positives to adjust for outcome measurement errors. A simulation study demonstrated that the new method enables unbiased estimation of hazard ratios from time-to-event data when the underlying sensitivity of outcome measurement and the false-positive rate are known. In practise, false-positive rates that are small in relation to the true hazard can be approximated to be zero without inducing bias. Moreover, the analysis of empirical data showed that the method is robust to misspecification of the sensitivity parameters as long as their ratio (${se}_{1}/{se}_{0}$) is set correctly and the cumulative risk of the true event is small.

We assumed the influenza vaccine’s mode of action is “leaky”, i.e. that vaccination provides only partial protection [12, 18, 19]. The appropriate effect measure of effectiveness is therefore the relative reduction in the infection hazard, assumed here to be constant over one influenza season. When estimating effectiveness based on the risk ratio, it has previously been shown that bias is determined by the ratio of the two sensitivity parameters [4]. We demonstrated that the same applies when effectiveness is based on the hazard ratio but only if the cumulative risk of the outcome is small, i.e. if the outcome is rare so that the risk set is largely unaffected by the occurrence of events.

Unlike in studies that exclusively refer to sensitivity as performance of a utilised laboratory test (e.g. [2]), register-based studies (e.g. [20]) use sensitivity in a broader sense as resulting from recording accuracy, healthcare seeking behaviour, swabbing policy and the sensitivity of diagnostic procedures. Based on a priori knowledge on surveillance practice in Finland and register data on laboratory-confirmed influenza, Shubin et al. [17] estimated that only 1 in 25 infections were ascertained and that the sensitivity varied across age, region and season. We expect that there are also differences by vaccination status but well-founded values for the sensitivity parameters ${se}_{0}$ and ${se}_{1}$ are not yet available. A study that closely follows a representative sample of the population through an influenza season and continuously validates the individuals’ infection status would be needed.

False-positive events result from diagnostic procedures with imperfect specificity. In register-based studies, the false-positive rate is additionally influenced by the accuracy of recording, sampling strategy, the rate of non-influenza but influenza-like illness, and healthcare seeking behaviour. Although the new method allows accounting for time-varying and differential occurrence of false positives (cf. Additional file 2: Web Appendix), the presented simulation study used constant false-positive rates corresponding to 2% or 16% of all recorded events in the unvaccinated being false-positive.

The simulation study results show that the impact of relatively small false-positive rates is negligible. Yang et al. [5] developed an expectation–maximisation algorithm to estimate $VE$ under non-specific observation of incident events when all true events are observed and validation data are available. Similarly to our method, their approach employs empirical hazards to address the problem of false positives. While Yang et al. allow repeated events, in our application the at-risk time is censored at the first recorded event. The data might then run short of true events if the false-positive rate is excessively high.

## Conclusion

The presented semiparametric method can be used to adjust for outcome measurement errors in the estimation of hazard ratios and effectiveness but requires specifying the sensitivity and the false-positive rate. In absence of exact information about these parameters, we consider our method as a tool for assessing the potential magnitude of bias given a range of parameter values, possibly stratified by appropriate covariates. The method would allow adjustment for confounders as in the PH model. Finally, although we considered an infectious disease epidemic, the applicability of the method is wider as long as the PH assumption holds.

## Supplementary Information

**Additional file 1.** R script. Description of data: The file provides four R functions and a working example (at the end of the script) allowing the realisation of the weighted partial likelihood approach and probabilistic deletion of false-positive events. sirdata() returns a piece-wise constant hazard mimicking the force of infection in a Susceptible-Infected-Removed epidemic. simdata() simulates time-to-event data and estdata() estimates the vaccine effectiveness and its standard error as described in the main text. outdata() combines the data simulation and estimation steps and returns the performance measures presented in the manuscript.

**Additional file 2.** Web Appendix. Description of data: Derivations of Eqs. (1, 2, 6 and 7). Simulation study set-up details. Additional file 2: Tables S1 and S2. Validity of the proportional hazards assumption. Additional file 2: Figure S1.

## Data Availability

The simulated datasets are available from the corresponding author on reasonable request.

## Abbreviations

CI: Confidence interval
SE: Standard error
VE: Vaccine effectiveness
