# Supplementary material for: Mitigation of biases in estimating hazard ratios under non-sensitive and non-specific observation of outcomes–applications to influenza vaccine effectiveness
Source: Emerg Themes Epidemiol. 2021 Jan 14;18:1. doi: 10.1186/s12982-020-00091-z (PMC7807790; doi:10.1186/s12982-020-00091-z)
Supplement: Supplementary file 2 — Additional file 2. Web Appendix. Description of data: Derivations of Eqs. (1, 2, 6 and 7). Simulation study set-up details. Additional file 2: Tables S1 and S2. Validity of the proportional hazards assumption. Additional file 2: Figure S1. [file 12982_2020_91_MOESM2_ESM.pdf]

Web Appendix for

Mitigation of biases in estimating hazard ratios under non-sensitive and non-specific observation of outcomes – applications to influenza vaccine effectiveness

by Ulrike Baum, Sangita Kulathinal, Kari Auranen

Page

1 Derivation of Equation (1) **2**

2 Derivation of Equation (2) **2**

3 Derivation of Equation (6) **3**

4 Derivation of Equation (7) **3**

5 Simulation study set-up details **4**

6 Table S1 **5**

7 Table S2 **6**

8 Validity of the proportional hazards assumption **7**

9 Figure S1 **8**

1 Derivation of Equation (1)

In absence of false-positive events ($\kappa(t) = 0$), a subject is observed to "survive" beyond time t if he/she escaped the true event through t or if the true event that occurred prior to or at time t is not detected due to imperfect sensitivity ($se < 1$), i.e.

$$\tilde{S}(t; se, \kappa(t) = 0) = \overbrace{S(t)}^{\text{no event}} + \overbrace{(1 - se)}^{\text{not detecting}} \overbrace{(1 - S(t))}^{\text{the true event}} = 1 - se \cdot \big(1 - S(t)\big). \tag{S.1}$$

If false-positive events occur at rate $\kappa(t)$, a subject is observed to "survive" beyond time t if he/she, in addition to the above conditions, also escaped any false-positive event through t, i.e.

$$\tilde{S}(t; se, \kappa(\cdot)) = [1 - se \cdot \big(1 - S(t)\big)] \cdot e^{-\int_0^t \kappa(u)\, du}. \tag{S.2}$$

Assuming the false-positive rate (κ) is constant, (S.2) simplifies to equation (1) in the main text:

$$\tilde{S}(t; se, \kappa) = [1 - se \cdot \big(1 - S(t)\big)] \cdot e^{-\kappa t}.$$

2 Derivation of Equation (2)

In general, the hazard $\lambda(t)$ is defined as the instantaneous event rate at time t given survival until t, i.e.

$$\lambda(t) = -\frac{S'(t)}{S(t)} = \frac{d}{dt}(-\log S(t)), \text{ where } S'(t) = \frac{d}{dt}S(t). \tag{S.3}$$

The cumulative hazard $\Lambda(t)$ is defined as

$$\Lambda(t) = \int_0^t \lambda(u)\, du,$$

which can alternatively be expressed as

$$\Lambda(t) = -\log(S(t)).$$

Applying the above equation to the observed cumulative hazard $\tilde{\Lambda}(t)$ and using (S.2), it follows that

$$\tilde{\Lambda}(t; se, \kappa(\cdot)) = -\log \tilde{S}(t; se, \kappa(\cdot)) = -\log \big[[1 - se \cdot \big(1 - S(t)\big)] \cdot e^{-\int_0^t \kappa(u)\, du}\big].$$

Subsequently,

$$\tilde{\lambda}(t; se, \kappa(\cdot)) = \frac{d}{dt}\tilde{\Lambda}(t; se, \kappa(\cdot)) = -\frac{\big[se \cdot S'(t) - \kappa(t) \cdot [1 - se \cdot \big(1 - S(t)\big)]\big] \cdot e^{-\int_0^t \kappa(u)\, du}}{[1 - se \cdot \big(1 - S(t)\big)] \cdot e^{-\int_0^t \kappa(u)\, du}}.$$

Based on (S.3) and (S.1), this simplifies to

$$\tilde{\lambda}(t; se, \kappa(t)) = \frac{se \cdot S(t) \cdot \lambda(t)}{1 - se \cdot \big(1 - S(t)\big)} + \kappa(t) = se \cdot \frac{S(t)}{\tilde{S}(t; se, \kappa(t) = 0)} \cdot \lambda(t) + \kappa(t).$$

Defining the weight $w(t)$ as

$$w(t) = \frac{S(t)}{\tilde{S}(t; se, \kappa(t) = 0)}, \qquad \text{(S.4)}$$

it follows that

$$\tilde{\lambda}(t; se, \kappa(t)) = se \cdot w(t) \cdot \lambda(t) + \kappa(t). \qquad \text{(S.5)}$$

Assuming the false-positive rate (κ) is constant, (S.5) simplifies to equation (2) in the main text:

$$\tilde{\lambda}(t; se, \kappa) = se \cdot w(t) \cdot \lambda(t) + \kappa.$$

3 Derivation of Equation (6)

Solving equation (1) in the main text for $S(t)$ gives

$$S(t) = 1 - (1 - \tilde{S}(t; se, \kappa) \cdot e^{\kappa t})/se. \qquad \text{(S.6)}$$

Using (S.6) with $\kappa = 0$, the weight (S.4) becomes

$$w(t) = \frac{1 - (1 - \tilde{S}(t; se, \kappa = 0))/se}{\tilde{S}(t; se, \kappa = 0)}.$$

Finally, using the Kaplan-Meier estimate ($\hat{\tilde{S}}(t)$) for $\tilde{S}(t)$ leads to the plug-in weight $\hat{w}(t)$ as in equation (6) in the main text:

$$\hat{w}(t) = \frac{1 - (1 - \hat{\tilde{S}}(t; se, \kappa = 0))/se}{\hat{\tilde{S}}(t; se, \kappa = 0)}.$$

4 Derivation of Equation (7)

According to (S.5), the observed hazard is the sum of the hazard of recording the true event and the false-positive rate. It follows that the probability $p(t)$ that an event observed at time t is a true event can be expressed by the ratio of the hazard of recording the true event to the observed hazard. Writing the hazard of recording the true event as a difference between the observed hazard and the false-positive

rate (cf. equation (2) in the main text) gives

$$p(t) = \frac{se \cdot w(t) \cdot \lambda(t)}{\tilde{\lambda}(t)} = \frac{\tilde{\lambda}(t) - \kappa(t)}{\tilde{\lambda}(t)}.$$

Assuming the false-positive rate (κ) is constant, this simplifies to equation (7) in the main text:

$$p(t) = \frac{\tilde{\lambda}(t) - \kappa}{\tilde{\lambda}(t)}.$$

5 Simulation study set-up details

A Susceptible-Infected-Removed epidemic was simulated using either reproduction number 1.15 and daily clearance rate 0.25 (cumulative risk of 0.25) or reproduction number 2.1 and daily clearance rate 0.066 (cumulative risk of 0.81). Each individual's true event time was sampled using piece-wise (weekly) constant approximations to $\lambda_0(t)$ and $\lambda_1(t)$ for the unvaccinated and vaccinated. Event times were rounded to the next full day. The approximation to probability $p(t)$ (cf. equation (8) in the main text) was applied separately for the unvaccinated and vaccinated. Instead of calculating the ratio of events to person-time using a daily time interval, the ratio was calculated as the sum of the events over a seven-day time window divided by the corresponding sum of person-time.

6 Table S1

Estimates of vaccine effectiveness (VE) under differential sensitivity and high cumulative risk of infection in absence of false-positive events. Mean of the vaccine effectiveness estimates ($\hat{VE}$), mean of the standard error estimates ($\hat{SE}$), standard error of the vaccine effectiveness estimates ($SE_{\hat{VE}}$), root-mean-squared error of the vaccine effectiveness estimates ($\sqrt{MSE}_{\hat{VE}}$), bias in percentage points, and empirical coverage probability (Cov) of the 95% confidence intervals when estimating vaccine effectiveness from 10^4 repeated data sets under differential sensitivity of 0.05 (se_0) and 0.03 (se_1) and a cumulative risk of 0.81 in the unvaccinated in absence of false-positive events. Naïve estimation was conducted under the incorrect assumption of perfect sensitivity ($se_0 = se_1 = 1$).

True	**Estimation adjusted for** $se_0 = 0.05$**,** $se_1 = 0.03$						**Naïve estimation**					
VE	$\hat{VE}$	$\hat{SE}$	$SE_{\hat{VE}}$	$\sqrt{MSE}_{\hat{VE}}$	Bias	Cov	$\hat{VE}$	$\hat{SE}$	$SE_{\hat{VE}}$	$\sqrt{MSE}_{\hat{VE}}$	Bias	Cov
Cohort of 50 000 individuals (30% vaccinated at season onset)												
**10%**	9%	0.05	0.11	0.11	−1	67%	43%	0.03	0.03	0.33	+33	0%
**30%**	30%	0.04	0.08	0.08	±0	72%	49%	0.03	0.03	0.20	+19	0%
**50%**	50%	0.03	0.06	0.06	±0	76%	58%	0.03	0.03	0.09	+8	17%
**70%**	70%	0.02	0.03	0.03	±0	83%	71%	0.02	0.02	0.02	+1	92%
**90%**	90%	0.01	0.01	0.01	±0	89%	89%	0.01	0.01	0.02	−1	88%
Cohort of 1 000 000 individuals (50% vaccinated at season onset)												
**10%**	10%	0.01	0.02	0.02	±0	66%	43%	0.01	0.01	0.33	+33	0%
**30%**	30%	0.01	0.02	0.02	±0	70%	49%	0.01	0.01	0.19	+19	0%
**50%**	50%	0.01	0.01	0.01	±0	73%	59%	0.01	0.01	0.09	+9	0%
**70%**	70%	0.00	0.01	0.01	±0	77%	71%	0.00	0.00	0.01	+1	25%
**90%**	90%	0.00	0.00	0.00	±0	85%	89%	0.00	0.00	0.01	−1	0%

7 Table S2

Estimates of vaccine effectiveness (VE) under differential sensitivity, small cumulative risk of infection and low rate of false-positive events. Mean of the vaccine effectiveness estimates ($\hat{VE}$), mean of the standard error estimates ($\hat{SE}$), standard error of the vaccine effectiveness estimates ($SE_{\hat{VE}}$), root-mean-squared error of the vaccine effectiveness estimates ($\sqrt{MSE}_{\hat{VE}}$), bias in percentage points, and empirical coverage probability (Cov) of the 95% confidence intervals when estimating vaccine effectiveness from 10^4 repeated data sets under differential sensitivity of 0.05 (se_0) and 0.03 (se_1) and a cumulative risk of 0.25 in the unvaccinated. The false-positive events occurred at rate $\kappa = 10^{-6}$ (per person-day) corresponding to a false-positive proportion of 2% among the unvaccinated. Naïve estimation was conducted under the incorrect assumptions of perfect sensitivity ($se_0 = se_1 = 1$) and absence of false positives ($\kappa = 0$).

True	**Estimation adjusted for** $se_0 = 0.05$, $se_1 = 0.03$, $\kappa = 10^{-6}$						**Naïve estimation**					
VE	$\hat{VE}$	$\hat{SE}$	$SE_{\hat{VE}}$	$\sqrt{MSE}_{\hat{VE}}$	Bias	Cov	$\hat{VE}$	$\hat{SE}$	$SE_{\hat{VE}}$	$\sqrt{MSE}_{\hat{VE}}$	Bias	Cov
Cohort of 50 000 individuals (30% vaccinated at season onset)												
**10%**	9%	0.10	0.12	0.12	−1	92%	45%	0.06	0.06	0.35	+35	0%
**30%**	29%	0.09	0.10	0.10	−1	93%	55%	0.05	0.05	0.26	+25	1%
**50%**	49%	0.07	0.08	0.08	−1	93%	67%	0.04	0.04	0.17	+17	7%
**70%**	69%	0.05	0.06	0.06	−1	94%	79%	0.03	0.03	0.10	+9	29%
**90%**	89%	0.03	0.03	0.03	−1	96%	92%	0.02	0.02	0.03	+2	80%
Cohort of 1 000 000 individuals (50% vaccinated at season onset)												
**10%**	10%	0.02	0.02	0.02	±0	92%	45%	0.01	0.01	0.35	+35	0%
**30%**	30%	0.02	0.02	0.02	±0	91%	56%	0.01	0.01	0.26	+26	0%
**50%**	50%	0.01	0.01	0.01	±0	93%	67%	0.01	0.01	0.17	+17	0%
**70%**	70%	0.01	0.01	0.01	±0	94%	79%	0.01	0.01	0.09	+9	0%
**90%**	90%	0.01	0.01	0.01	±0	94%	92%	0.00	0.00	0.02	+2	0%

8 Validity of the proportional hazards assumption

By analogy with the standard partial likelihood, the weighted partial likelihood (equation (4) in the main text) relies on assuming proportionality of the true hazards between the two comparison groups. One method to check for proportional hazards is to apply the log-log transformation to the true survival functions (andfleisch and Prentice, 2002). If the proportionality assumption holds, then

$$\log\left[-\log\left(S_1(t)\right)\right]-\log\left[-\log\left(S_0(t)\right)\right]=\log\left(1-VE\right).$$

In practise, the estimates $\hat{S}_0(t)$ and $\hat{S}_1(t)$ are plugged in. A linear relationship between the log-log transformed survival functions $\hat{S}_0(t)$ and $\hat{S}_1(t)$ supports the proportional hazards assumption. $\hat{S}_0(t)$ and $\hat{S}_1(t)$ can each be estimated solving (S.2) for $S(t)$ and plugging in the Kaplan-Meier estimate $\hat{\hat{S}}_0(t)$ or $\hat{\hat{S}}_1(t)$, respectively, i.e.

$$\hat{S}_v(t)=1-\frac{1-\hat{\hat{S}}_v(t)\cdot e^{-\int_0^t \kappa(u)\,du}}{se_v},\ v\in\{0,1\}.$$

Additionally, it may be noted that a non-parametric and time-dependent estimate of VE, which varies around the true value of VE, follows from the difference

$$\log\left[-\log\left(\hat{S}_1(t)\right)\right]-\log\left[-\log\left(\hat{S}_0(t)\right)\right]$$

if the proportionality assumption holds.

Kalbfleisch, JD and Prentice, RL. The statistical analysis of failure time data. 2nd ed. Hoboken: John Wiley and Sons, Inc. **2002**.

9 Figure S1

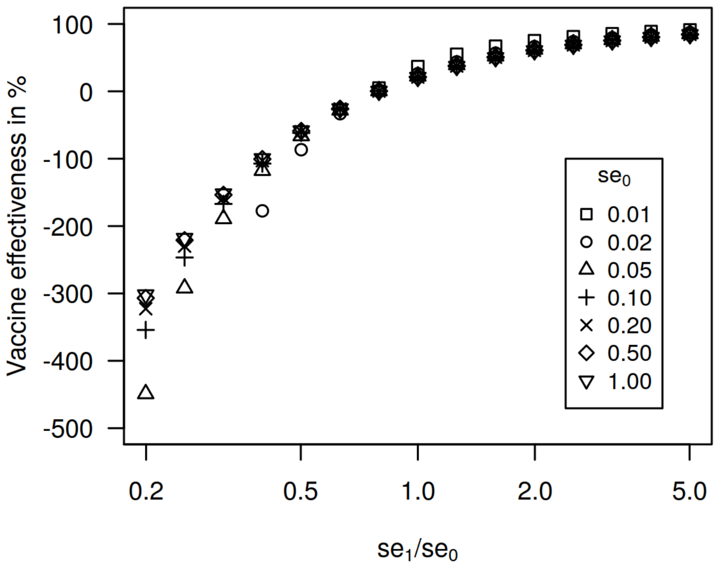

Estimates of influenza vaccine effectiveness in the Finnish elderly (N=1,160,986) in 2016/17.

Dependence of vaccine effectiveness estimates at $t = 196$ (days) on the assumed values of se_0 (symbols) and ratio se_1/se_0 (horizontal axis) based on equation (4) in the main text.
